# LONG-TERM OUTCOMES OF THE BENEFIT-FINDING GROUP INTERVENTION FOR ALZHEIMER’S FAMILY CAREGIVERS: A DOUBLE-BLIND RCT

**DOI:** 10.1093/geroni/igz038.2504

**Published:** 2019-11-08

**Authors:** Sheung-Tak Cheng

**Affiliations:** The Education University of Hong Kong, Hong Kong, Hong Kong

## Abstract

This study examines the long-term effects of benefit-finding on caregivers’ depressive symptoms (primary outcome), and global burden, role overload, and psychological well-being (secondary outcomes). 96 Hong Kong Chinese caregivers of relatives with Alzheimer’s disease were randomly assigned to receive the benefit-finding intervention (BFT) or one of two control conditions, namely, simplified psychoeducation (lectures only; SIM-PE) or standard psychoeducation (STD-PE). Caregivers received four biweekly one-to-one interventions of three hours each at their own homes. Participants and raters were blind to experimental assignment. We focused on outcomes measured at 4- and 10-month follow-ups. The trajectories of intervention effects were modeled by BFT x time and BFT x time2 interaction terms. Mixed-effects regression showed significant BFT x time2 interaction effects on depressive symptoms against both control conditions, suggesting diminishing BFT effects over time. Z tests showed that, compared with controls, BFT participants reported substantial reductions in depressive symptoms at 4-month follow-up (d = -0.85 and -0.75 vs. SIM-PE and STD-PE respectively). At 10-month follow-up, BFT was indistinguishable from STD-PE whereas a moderate effect was observed in the comparison with SIM-PE (d = -0.52). In addition, some inconsistent effects on role overload were observed but no effect was found for the other outcome variables. It is concluded that benefit-finding is an efficacious intervention for depressive symptoms in Alzheimer caregivers, with strong effects in the medium-term post-intervention and possible moderate effects in the long-term.

